# FGF-induced Pea3 transcription factors program the genetic landscape for cell fate determination

**DOI:** 10.1371/journal.pgen.1007660

**Published:** 2018-09-06

**Authors:** Ankur Garg, Abdul Hannan, Qian Wang, Tamica Collins, Siying Teng, Mukesh Bansal, Jian Zhong, Keli Xu, Xin Zhang

**Affiliations:** 1 Departments of Ophthalmology, Pathology and Cell Biology, Columbia University, New York, NY, United States of America; 2 Department of Ophthalmology, First Hospital of Jilin University, Changchun, Jilin, China; 3 PsychoGenics Inc., Tarrytown, New York, United States of America; 4 Burke Medical Research Institute, Feil Family Brain and Mind Research Institute, Weill Cornell Medicine, White Plains, NY, United States of America; 5 Cancer Institute, University of Mississippi Medical Center, Jackson, MS, United States of America; National Cancer Institute, UNITED STATES

## Abstract

FGF signaling is a potent inducer of lacrimal gland development in the eye, capable of transforming the corneal epithelium into glandular tissues. Here, we show that genetic ablation of the Pea3 family of transcription factors not only disrupted the ductal elongation and branching of the lacrimal gland, but also biased the lacrimal gland epithelium toward an epidermal cell fate. Analysis of high-throughput gene expression and chromatin immunoprecipitation data revealed that the *Pea3* genes directly control both the positive and negative feedback loops of FGF signaling. Importantly, *Pea3* genes are also required to suppress aberrant Notch signaling which, if gone unchecked, can compromise lacrimal gland development by preventing the expression of both *Sox* and *Six* family genes. These results demonstrate that Pea3 genes are key FGF early response transcriptional factors, programing the genetic landscape for cell fate determination.

## Author summary

FGF signaling regulates cell fate decision by inducing genome-wide changes in gene expression. We identified Pea3 family transcription factors as the key effectors of FGF signaling in reprograming the epithelia transcriptome. Pea3 factors control both the feedback and feedforward circuities of FGF signaling in lacrimal gland development. They also activate specific expression of Six and Sox family genes and suppress aberrant activation of Notch signaling. In the absence of Pea3 genes, the lacrimal gland progenitors become epidermal-like in their gene expression patterns. The study of Pea3 function resolves the long standing conundrum of how FGF induces the lacrimal gland fate, providing direction for regenerating the lacrimal gland to treat dry eye diseases.

## Introduction

During development of a complex multicellular organism, organ identity is determined by the combination of lineage-specific and signal-induced transcription factors. In mammalian lacrimal gland development, the extracellular signals include Fibroblast Growth Factor (FGF), Bone Morphogenetic Protein (BMP), Notch and Wnt that either cooperate or antagonize each other during budding, elongation and branching morphogenesis [1]. In particular, genetic evidence has revealed that FGF signaling initiated by the binding of FGF10/Fgf10, sent from the periocular mesenchyme, to FGFR2B/Fgfr2b on the conjunctival epithelium is indispensable for lacrimal gland development in both human and mouse [2–5]. Demonstrating the striking potency of FGF signaling in driving the lacrimal gland fate, ectopic expression of either rat *Fgf10* or human *FGF7* in the lens led to the formation of lacrimal gland-like cells in an area that under normal physiological conditions develops into the planar corneal epithelium [6, 7]. This is at least partly mediated by both the FGF-induced Sox9 expression required for lacrimal gland induction and the Sox10 expression for acini formation [8]. However, unlike BMP, Notch and Wnt which have well established downstream transcription effectors Smad, NICD and β-catenin, respectively, how FGF signaling triggers its transcriptional responses in lacrimal gland cell fate determination is not known.

The Pea3 family of transcription factors, composed of Pea3 (Etv4), Erm (Etv5) and Er81 (Etv1), are E26 transformation-specific (ETS)-domain proteins that can be phosphorylated by Mitogen-Activated Protein Kinase (MAPK) to control their subcellular localization, DNA binding and transactivation [9]. They have been shown to act as oncogenes in melanoma, breast, lung and prostate cancer, mimicking the aberrant activation of RAS-MAPK pathways commonly present in a multitude of malignancies [10]. During embryonic development, expression of the *Pea3* genes closely correlates with the activities of FGFs, making these genes suitable candidates for being the downstream effectors of FGF-Ras-MAPK signaling [11, 12]. Indeed, conditional inactivation of *Pea3*/*Erm* in the lung epithelium disrupted the Fgf10-Shh feedback loop, resulting in smaller lung sizes, but mice were grossly healthy and exhibited normal life-span [13, 14]. In the limb buds, Pea3 and Erm mediate FGF signaling in the proximal-distal (P-D) and anterior-posterior (A-P) patterning, which was evident by the growth retardation and mild polydactyly in the *Pea3*/*Erm* mutants [15, 16]. Nevertheless, these *Pea3*/*Erm* mutant phenotypes were relatively modest compared to the FGF signaling mutants in the same tissues.

In this study, we show that the MAPK-regulated Pea3 family of transcription factors are critical for lacrimal gland duct elongation and branching. Deletion of all three *Pea3* genes from the lacrimal gland epithelium resulted in ectopic expression of epidermal markers, shifting the lacrimal gland progenitor cells toward a cutaneous cell fate. In addition to previously reported FGF signaling response genes, we also identify *Six1* and *Six2* as being novel targets of the FGF-Pea3 axis, showing that these two genes cooperate in regulating lacrimal gland branching. Loss of *Pea3* results in aberrant upregulation of Notch signaling in the lacrimal gland primordia driven by Jag1-mediated lateral activation and concurrent downregulation of the Notch modulator, lunatic fringe. Aberrant Notch signaling sustains this auto-stimulatory loop by upregulating Jag1 expression, leading to the downregulation of FGF signaling effector genes and failure of lacrimal gland induction. The shift of cellular identity and discordance of FGF-Notch crosstalk in the absence of Pea3 transcription factors establishes *Pea3* genes as cell fate determinants in lacrimal gland development.

## Results

### Lacrimal gland development requires Pea3 transcription factors

**Fig 1:**
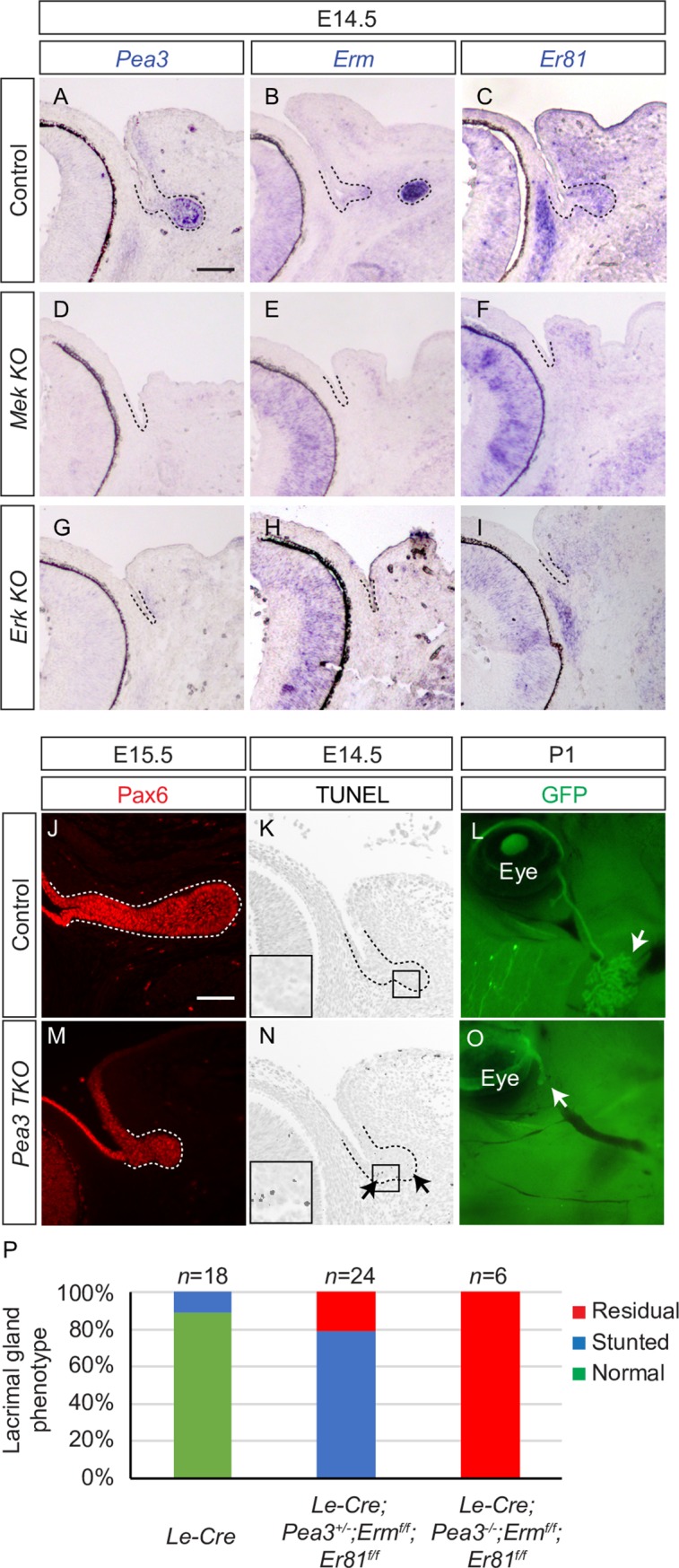
MAPK-regulated Pea3 transcription factors are required for lacrimal gland development. (**A-I**) Inactivation of Mek and Erk signaling abolished expression of the Pea3 family of transcription factors, *Pea3*, *Erm* and *Er81*, in the E14.5 lacrimal gland epithelium. (**J, M**) Lacrimal gland specific ablation of *Pea3/Erm/Er81* genes resulted in a smaller lacrimal gland bud as indicated by Pax6 expression. The lacrimal gland buds are marked by dotted lines. (**K, N**) TUNEL staining indicated an increase in apoptosis in the *Pea3 TKO* lacrimal gland bud (arrows). (**L, O**) At the P1 stage, *Pea3 TKO* mutants displayed a residual lacrimal gland as shown by the GFP reporter. (**P**) Quantification of the lacrimal gland phenotype in *Pea3 TKO* mutants at P1. Scale bars, 50μm.

Mouse lacrimal gland development commences at E13.5 with the thickening of the conjunctival epithelium, which subsequently forms a bud, entering the surrounding periocular mesenchyme by E14.5. This process is triggered by the mesenchymal release of Fgf10 which activates FGF signaling in the epithelium. This signaling leads to the activation of the Pea3 family of ETS transcription factors, *Pea3*/*Etv4*, *Erm*/*Etv5*, *Er81*/*Etv1* (Fig 1A–1C, dotted lines) [5, 17, 18]. We conditionally deleted *Mek* and *Erk* using an *Le-Cre* transgenic mouse line, in which Cre-recombinase linked to an IRES-GFP reporter was expressed in the conjunctival epithelium and the lacrimal gland [19]. In both *Le-cre; Mek1*^*fl/fl*^*; Mek2*^*-/-*^ (*Mek KO*) and *Le-cre; Erk1*^*-/-*^*; Erk2*^*fl/fl*^ (*Erk KO*) lacrimal gland epithelia, expressions of *Pea3* transcription factors were abolished (Fig 1D–1I, dotted lines).

To study the function of these transcription factors, we conditionally deleted *Erm* and *Er81*, two members of the Pea3 family of transcription factors, in a *Pea3*-null background using *Le-Cre*. Indicated by the lacrimal gland progenitor cell marker Pax6, the lacrimal gland primordia in E15.5 *Le-cre; Pea3*^*-/-*^*; Erm*^*fl/fl*^*; Er81*^*fl/fl*^ (hereafter referred to as *Pea3 TKO*) embryos were noticeably smaller in size compared to the control (Fig 1J and 1M, dotted lines). As reflected by TUNEL staining, this was consistent with an increase in apoptosis seen in the lacrimal gland primordia (Fig 1K and 1N, arrows). Analysis of the malformed gland marked by GFP expression additionally showed that both duct elongation and branching were severely compromised at the post-natal P1 stage (Fig 1L and 1O, arrows). In contrast, the lacrimal gland phenotype was considerably less severe in mice carrying at least one normal copy of *Pea3* (Fig 1P), indicating the importance of *Pea3* gene. Of note, unlike *Mek* and *Erk KO* that displayed complete lacrimal gland aplasia, *Pea3 TKO* still presented with residual lacrimal glands. These results suggest that Pea3 transcription factors mediate some but not all of MAPK-dependent processes in lacrimal gland development.

### Pea3 transcription factors fine tune FGF signaling

**Fig 2:**
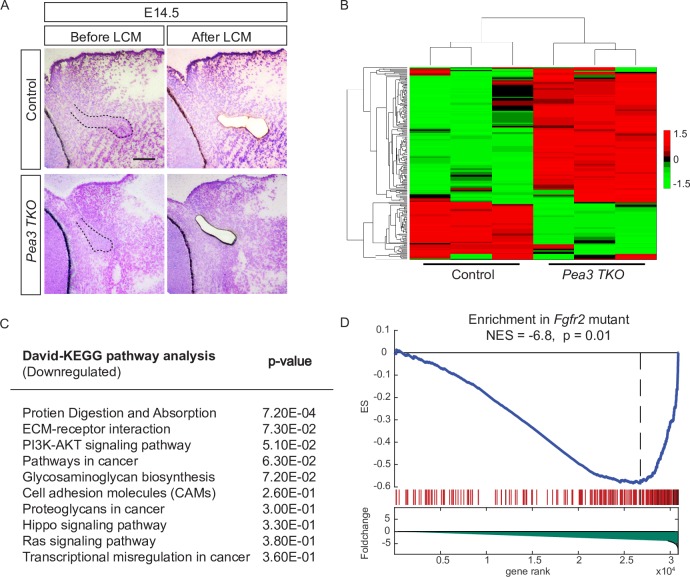
Bioinformatics analysis indicates that Pea3 transcription factors are downstream of FGF signaling during lacrimal gland development. (**A**) Images of sections before and after laser capture microscopy to isolate the lacrimal gland bud. Scale bar, 50μm. (**B**) Clustergram analysis of the top 200 differentially expressed genes in RNA-seq data from 6 different samples (Control, *n* = 3, Mutant, *n* = 3). The control and mutant samples were segregated into two separate clusters, indicating the robustness of our data (r = 0.8). (**C**) KEGG pathway analysis of downregulated genes in *Pea3* mutant using DAVID indicated that several key pathways were significantly downregulated. (**D**) Gene set enrichment analysis (GSEA) analysis showed that the transcriptome changes in *Pea3* mutants resemble that of the previously reported *Fgfr2* mutant.

In order to decipher the gene regulatory network of Pea3 transcription factors, E14.5 lacrimal gland epithelial tissue from control (*Le-Cre*) and mutant (*Pea3 TKO*) mouse embryos were micro-dissected using laser capture microscopy and subjected to RNA-seq (Fig 2A, *n* = 3 per condition). Unsupervised clustering analysis of the normalized data revealed that control and mutant samples were separated into two distinctive groups and that data from individual samples within each group were highly correlated (Fig 2B, r = 0.8), indicating the robustness of the obtained results. Gene ontology analysis showed that biological processes such as protein degradation, ECM interaction, glycosaminoglycan biosynthesis and cell adhesions are significantly downregulated in *Pea3 TKO* mutants (Fig 2C), which is in line with the previous findings that proteoglycans and ECM proteins play important roles in lacrimal gland development [5, 8, 17, 18]. In addition, PI3K and Ras pathways were also impaired in *Pea3 TKO* mutants, suggesting that downstream effectors of FGF signaling may also be compromised. To validate this idea, we compared our dataset with the previously published result from the *Fgfr2* conditional knockout [8]. Gene set enrichment analysis (GSEA) revealed that there was indeed a significant overlap in downregulated genes between *Pea3 TKO* and *Fgfr2* mutants (NES = -6.8, p = 0.01) (Fig 2D) [20]. Taken together, these results are consistent with the notion that the *Pea3* family of genes act downstream of the FGF signaling cascade.

**Fig 3:**
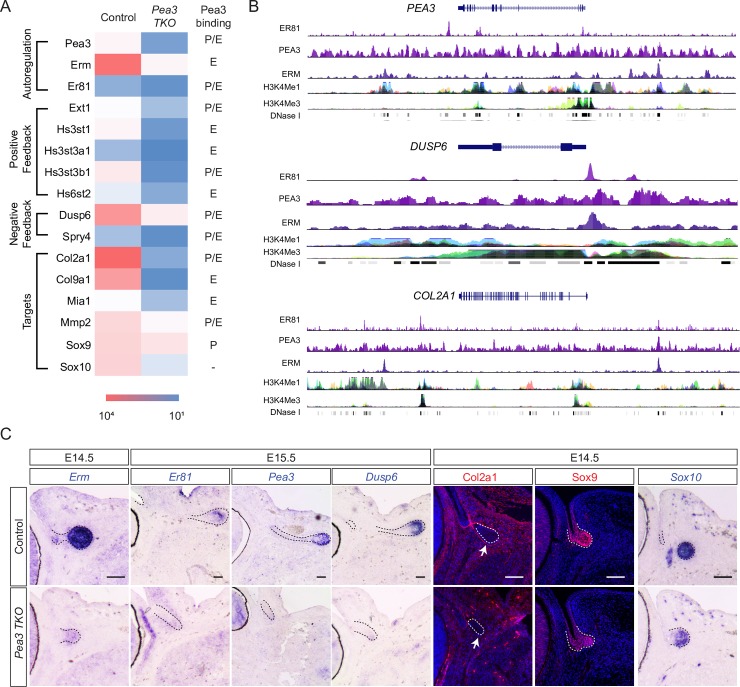
Pea3 induces both the feedback and feedforward circuits in FGF signaling. (**A**) The heatmap of Pea3-dependent genes involved in feedforward and feedback regulation of FGF signaling. P: promoter region, E: enhancer region. (**B**) Analysis of ChIP-seq data in the LoVo and GIST48 cancer cells line showed binding sites for the Pea3 transcription factors in the promoter and enhancer regions of *Pea3*, *Dusp6* and *Col2a1*. (**C**) The Pea3-dependent genes confirmed by RNA in situ hybridization and immunostaining in the lacrimal gland bud (dotted lines). Arrows indicate the basement membrane staining of Col2a1. Scale bars, 50μm.

Further analysis revealed that Pea3 transcription factors were uniquely positioned to fine tune the FGF signaling outcome. First, Pea3 transcription factors promoted their own expressions in the lacrimal gland bud, as *Pea3*, *Erm* and *Er81* transcripts were reduced in *Pea3 TKO* RNA-seq dataset (Fig 3A). Second, expression of heparan sulphate biosynthetic enzymes Ext1, Hs3st and Hs6st was also down regulated (Fig 3A). Since heparan sulphate proteoglycans are known to act as co-receptors for Fgf10, this was expected to dampen the positive feedback mechanism of FGF signaling. Third, Pea3 transcription factors were required for the expression of Dusp6 and Spry4 (Fig 3A), which are both inhibitors of Ras-MAPK signaling. Reevaluating the available ChIP-seq data from the human LoVo and GIST48 cancer cell lines [21, 22], we found that all of the above negative and positive feedback genes could be bound by either PEA3, ERM or ER81 in their promoter (within 5000 bp of the transcriptional start site) and/or enhancer regions (beyond 5000 bp upstream or downstream to the promoter site) (Fig 3A and 3B). Many of the ChIP-seq peaks for PEA3 proteins overlapped with H3K4Me1, H3K4Me3 and DNAse I sensitivity sites, signifying an open chromatin conformation in these regions (Fig 3B). Since *Erm* was the most highly expressed Pea3 transcription factor during lacrimal gland development (Fig 1B), we searched for the putative Erm binding sites in the corresponding mouse genomic regions using TRANSFAC database (S1 Fig). By chromatin immunoprecipitation, we confirmed that Erm protein indeed bound to the *Ext1*, *Dusp6*, *Col2a1* and *Mmp2* loci in P4 lacrimal gland cells (S1 Fig). In addition, RNA in situ hybridization experiments confirmed that *Pea3*, *Erm*, *Er81* and *Dusp6* genes were down regulated specifically in the *Pea3 TKO* lacrimal gland primordia (Fig 3C). These data show that Pea3 transcription factors play a central role in modulating the levels of FGF signaling by regulating the positive and negative feedback loops involved in the fine tuning of this pathway.

### The lacrimal gland progenitors were biased toward the epidermal fate in *Pea3* mutants

The Sox family of transcription factors Sox9 and Sox10 have been previously identified as downstream targets of FGF signaling important for lacrimal gland development [8]. The expression levels of *Sox10* were severely diminished in *Pea3 TKO* mutants, whereas the reduction in Sox9 expression was less dramatic (Fig 3A and 3C). Interestingly, the ChIP-seq analysis suggested that the promoter of *SOX9* but not that of *SOX10* harbored direct binding sites for PEA3 factors (Fig 3A). Sox9 was previously shown to regulate the expression of extracellular matrix related genes *Col2a1*, *Col9a1*, *Mia1* and *MMP2*, which is consistent with the dynamic remodeling of the extracellular matrix during lacrimal gland development [8]. Interestingly, these genes were also occupied by PEA3 transcription factors in their promoter/enhancer regions in GIST48 and LoVo cells, with their expressions being down regulated in *Pea3 TKO* mutants (Fig 3A–3C). Therefore, by controlling both Sox9 and its downstream targets, Pea3 transcription factors activate a feedforward mechanism in regulating lacrimal gland development.

**Fig 4:**
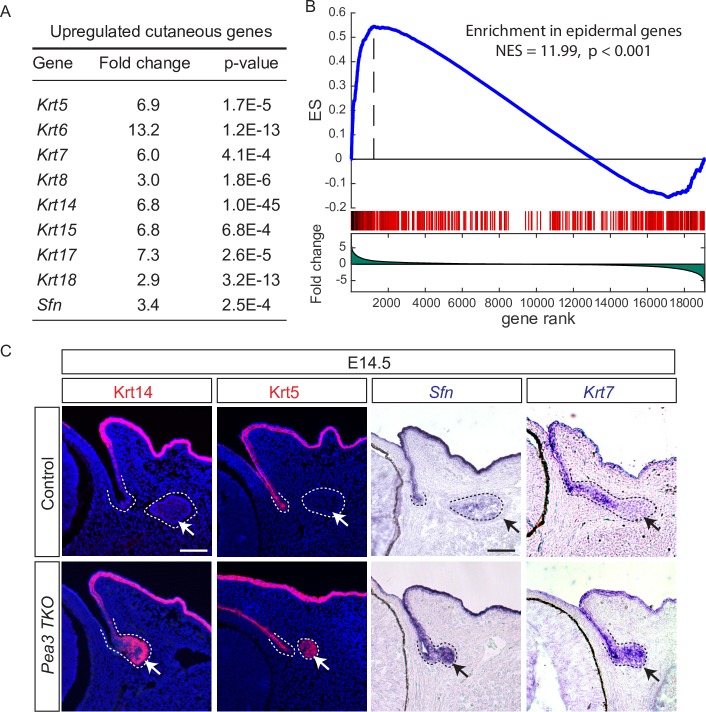
Loss of *Pea3* disrupted the adoption of the lacrimal gland fate. (**A**) A list of genes typically expressed in the cutaneous epithelium that were upregulated in the *Pea3 TKO* lacrimal gland bud. (**B**) GSEA analysis of differentially upregulated genes in the *Pea3 TKO* mutants showed a strong resemblance to the epidermal gene signature. (**C**) Ectopic expression of Krt14, Krt5, Sfn and Krt7 in the *Pea3* mutant lacrimal gland. Scale bars, 50μm.

Strikingly, the transcriptome analysis additionally revealed that many keratin genes were upregulated in *Pea3 TKO* mutants (Fig 4A). This result was especially unexpected because the keratins that were ectopically expressed are typically found in the cutaneous epithelium during embryonic development, rather than in the lacrimal gland. This led us to hypothesize that there was a shift in cell identity from the lacrimal gland fate to the epidermal-like fate in the absence of *Pea3* genes. To test this idea, we performed GSEA of differentially upregulated genes in *Pea3 TKO* mutants compared to the published gene expression datasets of E14.5 mouse embryonic skin [23]. This analysis showed that the transcriptome of the *Pea3 TKO* lacrimal gland primordia was significantly enriched in genes prevalent in the epidermis (Fig 4B, NES = 11.99, p<0.001) and hair follicle placode (NES = 9.0, p<0.01). In contrast, no significant similarities were observed when compared with the dermal condensates, skin fibroblast, melanocyte or Schwann cells. We next examined a set of genes that displayed nested expressions from the epidermis, to the conjunctiva to the lacrimal gland. At E14.5, Krt14 was mostly restricted to the epidermis, and Krt5 and *Sfn* were only present in the skin epidermis and the conjunctival epithelium, whereas *Krt7* expression was expanded into the stalk region of the lacrimal gland but excluded from the bud (Fig 4C). In the *Pea3 TKO* mutant, all these genes were expressed in the lacrimal gland primordia. These data indicated that Pea3 proteins prevented the lacrimal gland progenitors from adopting the epidermal fate.

### Six1 and Six2 cooperate to regulate branching morphogenesis downstream to FGF-Pea3 signaling

**Fig 5:**
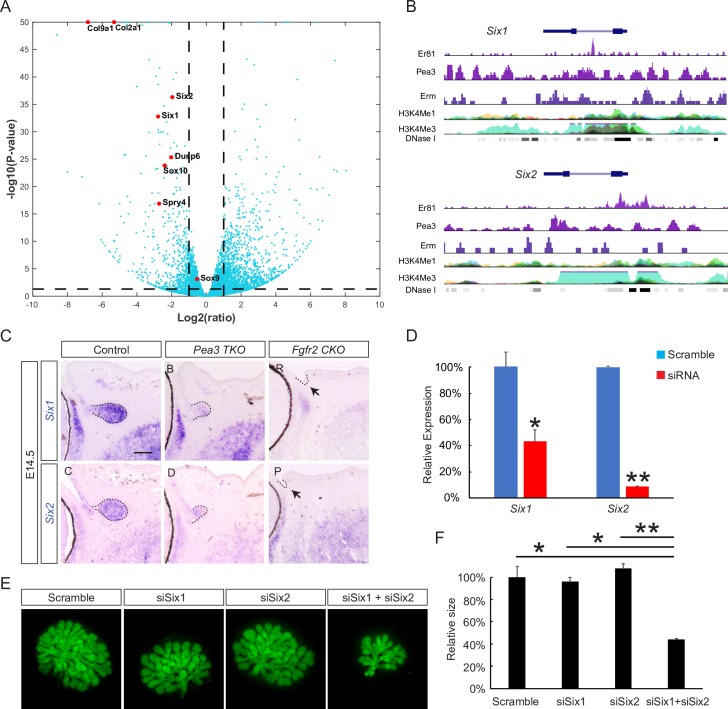
*Six1* and *2* are downstream targets of Pea3. (**A**) Volcano plot of dysregulated genes in *Pea3* mutant lacrimal gland buds. Red dots highlight *Six1*, *Six2* and other known targets of FGF signaling down regulated in *Pea3* mutants. (**B**) Pea3 binding sites were presented in the *Six1* and *Six2* loci as shown by analysis of ChIP-seq datasets. (**C**) Both *Six1* and *2* were downregulated in *Pea3* and *Fgfr2* mutants. Scale bar, 50μm. (**D**) Quantitative-PCR demonstrated that siRNA reduced the expressions of *Six1* and *Six2*. Student’s t test, *P<0.01, **P<0.0001, n = 3. (**E-F**) siRNA knockdown of *Six1* and *Six2* synergistically disrupted lacrimal gland development in explant lacrimal gland cultures (Mean lacrimal gland sizes relative to control + s.e.m. are shown. One-way ANOVA test, *P<0.05, **P<0.02, n = 3 for each condition).

To further understand the molecular mechanism of *Pea3* mutant defects, we sought to determine the most differentially regulated genes in our dataset. For this analysis, the gene expression changes depicted by Log_2_ (fold change) were plotted on the x-axis against the corresponding statistical significance depicted by -Log_10_ (p-value) on the y-axis (Fig 5A). Apart from the aforementioned FGF-responsive genes *Spry4*, *Dusp6*, *Col2a1*, *Col9a1*, *Sox9 and Sox10*, transcription factors *Six1* and *Six2* also emerged as significantly downregulated genes in *Pea3 TKO* mutants. Importantly, both *SIX1* and *SIX2* loci in GIST48 and LoVo cells displayed significant ChIP-seq peaks for PEA3, Erm and ER81 in open chromatin conformations marked by histone H3K4Me1 and H3K4Me3 methylations and DNase I sensitivity, suggesting they could be direct targets of PEA3 transcription factors (Fig 5B). Indeed, in situ hybridization for *Six1/ Six2* revealed that their expressions were significantly reduced in E14.5 *Pea3 TKO* lacrimal glands (Fig 5C, dotted lines) and abolished in *Le-Cre; Fgfr2*^*fl/fl*^ mutants (Fig 5C, arrows). Therefore, *Six1* and *Six2* are transcriptional targets of Pea3 and FGF signaling in the lacrimal gland epithelium.

While a *Six1* deletion has been shown to affect lacrimal gland duct elongation and branching [24], a *Six2* mutant phenotype hasn’t previously been reported. We examined lacrimal gland development in *Six2* knockout embryos at E15.5, but did not observe any gross abnormalities (S2 Fig). This could be due to compensation by *Six1* during lacrimal gland development. To test this idea, we used siRNAs against *Six1* and *Six2* genes, which resulted in significant down regulation of their expressions in a cell based assay (Fig 5D). In the ex-vivo lacrimal gland culture, exogenous Fgf10 induced significant growth of the E17.5 lacrimal gland primordia, which was dampened by siSix1 but not by scrambled siRNA (Fig 5E and 5F). Although siSix2 did not display any effect, combined application of siSix1 and siSix2 led to significant reduction in the size of lacrimal gland buds induced by Fgf10. These results show that Six1 and Six2 act synergistically to regulate lacrimal gland development.

### Pea3 transcription factors suppressed Notch signaling to promote lacrimal gland induction

**Fig 6:**
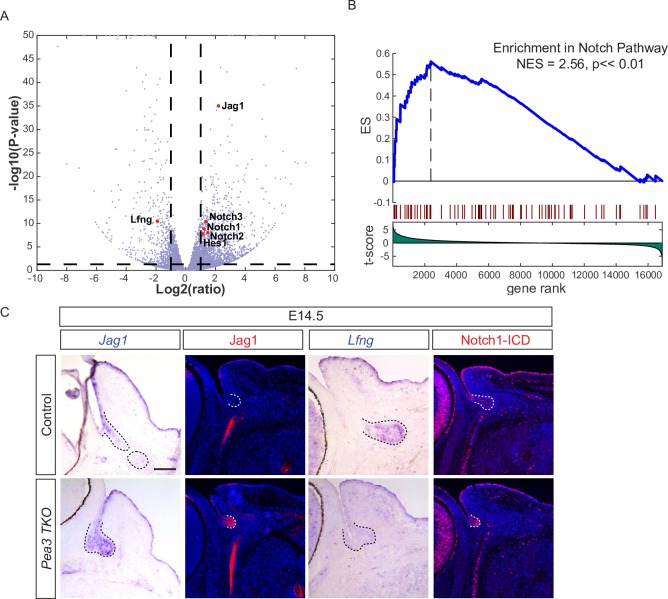
Notch signaling was induced in the *Pea3* mutant lacrimal gland. (**A-B**) GSEA analysis and Volcano plot data revealed that Notch pathway genes were enriched in *Pea3* mutants. (**C**) The *Pea3* knockout resulted in loss of the Notch regulator *Lfng*, induction of ligand Jag1 and ectopic expression of the effector, Notch-ICD, in the lacrimal gland bud (dotted lines). Scale bar, 50μm.

Although Pea3 proteins generally function as transcriptional activators, they can also act as repressors in certain contexts [25], thus we examined genes upregulated in the *Pea3 TKO* mutants. Notably, pathway analysis revealed activation of the Notch signaling pathway reflected by an increase in expression of the ligand Jag1, receptors Notch1, Notch 2 and Notch3, downstream target Hes1 and a reduced expression of *Lunatic fringe* (*Lfng*) (Fig 6A). This was further confirmed by GSEA of Notch signaling genes in the *Pea3 TKO* transcriptome (Fig 6B). Indeed, RNA in situ hybridization showed that *Jag1* mRNA was normally restricted to the surface ectoderm and conjunctiva at E14.5, but in the *Pea3 TKO* mutants, *Jag1* transcripts were ectopically expressed in the lacrimal gland primordia, with its translated protein form being prominently induced in the same area (Fig 6C). This was in sharp contrast to *Lfng*, a gene that was readily detectable in the control lacrimal gland with its expression being significantly reduced in the *Pea3 TKO* mutants (Fig 6C). In line with these findings, *Pea3 TKO* mutant lacrimal gland primordia displayed readily detectable staining patterns of Notch1 intracellular domain (Notch1-ICD), demonstrating that Notch signaling was aberrantly activated.

**Fig 7:**
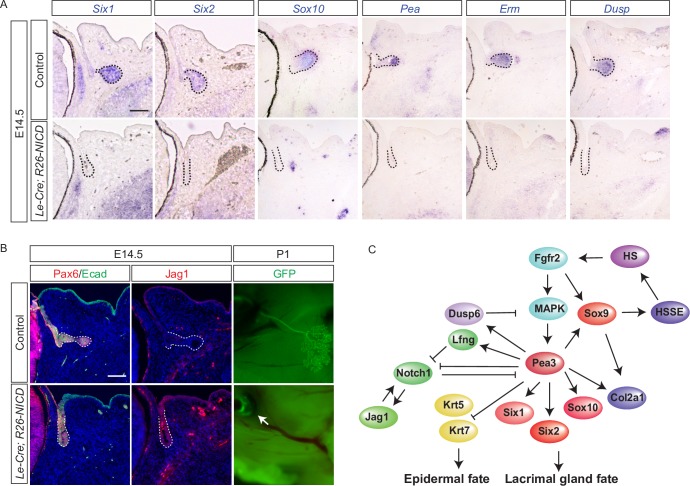
Ectopic activation of Notch signaling abolished lacrimal gland development. (**A**) Ectopic induction of Notch signaling by Notch-ICD overexpression abrogated many of the *Pea3*-regulated genes in the lacrimal gland bud (dotted lines). Scale bar, 50μm. (**B**) Notch-ICD overexpression did not perturb lacrimal gland progenitor cell markers Pax6 and Ecad but resulted in the up regulation of Jag1 expression. At P1, no lacrimal gland was observed (arrow). Scale bar, 50μm. (**C**) Schematic diagram of the *Pea3*-regulated genetic network in lacrimal gland development.

After establishing the misplaced activation of Notch signaling in the lacrimal gland, we subsequently investigated the functional significance of its activation in this developing tissue. Lfng is a glycosyl transferase that prevents Jag1-mediated Notch signaling in a context dependent manner [26–28]. Consistent with its negative role in Notch signaling, genetic ablation of *Lfng* resulted in a moderate increase in Notch1-ICD staining in the tip of the E14.5 lacrimal gland (S3A–S3D Fig). Interestingly, lacrimal gland size was reduced in P10 *Lfng* knockout pups, suggesting that the loss of *Lfng* expression likely contributed to the *Pea3 TKO* lacrimal gland phenotype (S3E and S3F Fig). Nevertheless, the *Lfng* knockout did not fully activate Notch signaling to the extent seen in the *Pea3 TKO* mutants. This prompted us to directly express the Notch1 intracellular domain in the developing lacrimal gland using the Cre-inducible *R26-N1CD* allele. In E14.5 *Le-Cre; R26-N1CD* embryos, expressions of *Six1* and *Six2* were lost, but the lacrimal gland progenitor cell markers Pax6 and E-cadherin were retained (Fig 7A and 7B, dotted lines). The downstream targets of FGF signaling such as *Sox10*, *Pea3*, *Erm* and *Dusp6* were also downregulated (Fig 7A). Interestingly, Jag1 was upregulated in the fornix of the conjunctiva where the lacrimal gland progenitors resided, suggesting that Notch signaling acted in an auto-stimulatory loop to increase Jag1 expression (Fig 7B, dotted lines). At P1, no lacrimal gland was found in *Le-Cre; R26-N1CD* embryos (Fig 7B, n = 10), demonstrating that aberrant activation of Notch was deleterious to lacrimal gland development.

## Discussion

FGF signaling plays an instructive role in lacrimal gland development, controlling its fate determination and morphogenesis. Mediated by the canonical Ras-MAPK pathway, FGF signaling induces expression of Pea3 transcription factors during the formation of both the epithelial and mesenchymal compartments of the lacrimal gland [18, 29]. In this study, we showed that Pea3 transcription factors were necessary to establish the identity of the lacrimal gland epithelium, turning it away from epidermal and conjunctival cell fates (Fig 7C). This was further attributed to the loss of *Six1* and *Six2* during lacrimal gland development, leading to both the disruption of duct elongation and branching morphogenesis. In addition, we found that *Pea3* transcription factors inhibited the Notch signaling pathway which, when activated, prevents the expression of *Six* and *Sox* genes and causes the abortion of lacrimal gland induction. Collectively, our data demonstrate that *Pea3* transcription factors control the expression profiles of key genes involved in the promotion of lacrimal gland identity and morphogenesis.

The regulatory mechanisms controlling the expression levels of *Six1* and *Six2* are not well understood. *Six1* deficiencies cause defects in organs that include the inner ear and kidney, both of which also develop through an epithelial-mesenchymal interaction like that which occurs in the lacrimal gland. However, contrary to what we observed in the lacrimal gland, analyses of inner ear development showed that Pea3 negatively regulated the pre-placodal genes *Six1* and *Eya2*, and *Six1* acted upstream of *Jag1* in the Notch signaling pathway [30, 31]. In kidney development, both Six1 and Six2 are expressed in the cap mesenchymal area where they are required for the ureteric budding and branching process [32–34]. Although Pea and Erm transcription factors are present in both the ureteric bud and the mesonephric mesenchyme, they are only required in the epithelial compartment to mediate Ret signaling [35]. *SIX1*/*Six1* have been previously implicated in lacrimal gland development in humans and in mice. A heterozygous missense mutation in the *SIX1* gene causes autosomal dominant lacrimal gland stenosis whereas *Six1* knockout mouse embryos displayed small lacrimal glands with duct elongation and branching defects [24]. Our RNA-seq analysis showed that *Six2* was expressed at 5.6 folds higher than *Six1* in the developing lacrimal gland epithelium, but surprisingly, *Six2* null mutant embryos did not exhibit any lacrimal gland phenotype. This was likely due to compensation by *Six1*, as our explant culture experiments showed that knockdown of both *Six1* and *Six2* synergistically disrupted branching morphogenesis of the lacrimal gland. We further showed that *Six1* and *Six2* were controlled by Pea3 transcription factors downstream of FGF signaling in the lacrimal gland epithelium. Thus, *Six1* and *Six2* genes are novel targets of Pea3 transcription factors in regulating lacrimal gland morphogenesis.

Although Notch activity is important for maintaining the postnatal homeostasis of the lacrimal gland [36], its temporal requirement during development has yet to be established. We have shown that Pea3 transcription factors prevent ectopic activation of Notch signaling during lacrimal gland induction. Analysis of our RNA-seq data for the modulators of Notch signaling showed that Lfng was the only Fringe family gene expressed abundantly in the lacrimal gland and its expression was significantly downregulated in the *Pea3 TKO* mutants. Lfng is a glycosyltransferase that adds O-linked fucose residues to the extracellular domain of the Notch receptor in order to modulate its ligand binding [37]. Lfng has been shown to potentiate the Dll-mediated but inhibit the Jag1-mediated Notch1 signaling pathways [26–28]. During sensory hair cell development in the inner ear, Lfng co-expresses with Jag1 and, when mutated in mice, partially rescues the *Jag2* knockout phenotype [38, 39]. During lacrimal gland development, Pea3 transcription factors may turn on the expression of Lfng to down regulate Jag1-mediated Notch signaling. This model was supported by the down regulation of *Lfng* levels in the absence of Pea3 transcription factors, and increased Notch1-ICD staining with reduced size of the lacrimal gland in the *Lfng* knockout. *Lfng* was not the only component of Notch signaling targeted by the Pea3 transcription factors in the lacrimal gland. In fact, the expression levels of *Jag1*, *Notch* and *Hes1* were all elevated in the *Pea3 TKO* mutants, resulting in a much higher level of Notch1-ICD staining compared to that seen in the *Lfng* mutants. To replicate such strong activation of Notch signaling, we directly expressed Notch1-ICD in the ocular surface, resulting in loss of both the *Six* and *Sox* genes and abrogation of lacrimal gland induction. These results highlight the importance of inhibiting aberrant premature Notch signaling during lacrimal gland development.

Our study revealed a previously underappreciated FGF signaling network systematized by the Pea3 transcription factors targeting *Sox*, *Six* and Notch signaling pathways during development of the lacrimal gland. We also showed that Pea3 transcription factors not only directly promote the expression of heparan sulfates involved in potentiating FGF signaling but also activate expression of the inhibitory factors Sprouty4 and Dusp6. We would like to suggest that by inducing both positive and negative feedback loops the Pea3 family of proteins may amplify the transcription response to low levels of FGF signaling but dampen the response to strong FGF signals. This non-linear transcriptional response mechanism can stabilize the FGF signaling network output given a wide range of FGF signal input, buffering the developmental system in face of environmental perturbations.

## Materials and methods

### Ethics statement

The animal experiments were approved by Columbia University Institutional Animal Care and Use Committee (Protocol number: AAAR0429).

### Mice

Mice carrying *Erk1*^*-/-*^, *Erk2*^*flox*^, *Lfng*^*-/-*^,*Mek1*^*flox*^ and *Mek2*^*-/-*^ alleles were bred and genotyped as described [28, 40, 41]. We obtained *Er81*^*flox*^ mice from Dr. Silvia Arber (University of Basel, Basel, Switzerland), *Pea3*^*-/-*^ and *Erm*^*flox*^ mice from Dr. Xin Sun (University of California at San Diego, San Diego, CA), *Fgfr2*^*flox*^ from Dr. David Ornitz (Washington University Medical School, St Louis, MO) and Le-Cre mice from Dr. Ruth Ashery-Padan (Tel Aviv University, Tel Aviv, Israel). [15, 19, 42, 43]. *Rosa-N1-ICD*^*flox/+*^ mice were obtained from Jackson lab (Stock # 008159). Animals were maintained in a mixed genetic background. Lacrimal gland growth and morphology were identical in *Le-Cre* and *Le-Cre;Pea3*^*+/-*^*;Erm*^*flox/+*^*;Er81*^*fl*ox/+^ mice, which were used as controls throughout the conducted experiments. Mice were housed in specific pathogen free (SPF) facility that employed a 12-hour light-dark cycle and were given standard mouse feed.

### RNA in situ hybridization

RNA in situ hybridization was performed as previously described [44]. Briefly, the mouse embryos were harvested, fixed overnight in 4% PFA, equilibrated in 30% sucrose and cryo-frozen in OCT. On the day of the experiment, OCT blocks were sectioned at 10 μm, hybridized with the diluted probe at 68°C overnight in a wet chamber and moistened with solution containing 50% Formamide and 1X Salt (0.2M NaCl, 10mM Tris, 5mM NaH_2_PO_4_, 5mM Na_2_HPO_4_, 5mM EDTA). The probe was diluted at 1:200–500 in a pre-warmed hybridization buffer and incubated at 70°C for at least 10 minutes. On the next day, slides were washed 3X in wash buffer (1X SSC (150mM NaCl, 15mM Sodium citrate, pH 7), 50% Formamide) at 68°C. After cooling, slides were washed 2X with MABT (100mM maleic acid, 150mM NaCl, pH 7.5, 0.1% Tween 20) and incubated at room temperature for 30 min. Slides were then blocked with 20% Sheep serum in MABT for 1 hour, followed by an overnight incubation with anti-DIG antibody (1:1500) at 4°C. On the next day, slides were washed 4-5X with MABT and 2X with alkaline phosphatase buffer. For color development, slides were covered with BM purple substrate and incubated at room temperature for 4–24 hrs. The following probes were used: *Pea3*, *Erm5* (from Dr. Bridget Hogan, Duke University Medical Center, Durham, NC, USA), *Er81* (from Dr. Gord Fishell, New York University Medical Center, New York, NY, USA), *Jag1* (from Dr. Doris Wu, National Institute on Deafness and Other Communication Disorders, National Institutes of Health, Bethesda, MD), *Lfng* (from Dr. Andy Groves, Baylor College of Medicine, Houston, TX), *Six1* (from Dr. Bernice Morrow, Albert Einstein College of Medicine, New York, NY, USA), *Six2* (from Dr. Thomas Caroll, UT Southwestern Medical center, Dallas, TX, USA), *Sox10* (from Dr. Anthony Firulli, Indiana University School of Medicine, Indianapolis, IN, USA), *Dusp6* (full length cDNA IMAGE clone: 3491528, Open Biosystems, Huntsville, AL, USA), *Sfn* and *Krt7* (full length cDNA IMAGE clone: 184592 and 40614, the DNA Resource Core, Harvard Medical School, Boston, MA, USA).

### Immunohistochemistry

For immunohistochemistry of paraffin samples, sections were deparaffinized and rehydrated by serial treatment with histosol followed by decreasing percentages of ethanol solutions [44, 45]. For cryosections, sections were briefly washed with PBS to remove OCT. Antigen retrieval was performed with microwave boiling in citrate buffer (10 mM sodium citrate, pH 6.0) for 1–2 minutes followed by heating for 10 minutes at a low power setting. Sections were then washed with PBS and blocked with 5% NGS/0.1% Triton in PBS. Primary antibody incubation was performed overnight at 4°C in a humid chamber followed by incubation with fluorescent-conjugated secondary antibodies for 1 hour at room temperature in the dark. For signal amplification, HRP-conjugated secondary antibodies were used, followed by washing and equilibration with TNT buffer. The slides were then incubated with Tyramide reagent for 10 minutes, washed with TNT buffer, stained with DAPI and mounted with anti-fade reagent, 0.2% NPG, 90% glycerol in 1X PBS. The following primary antibodies were used: Pax6 (PRB-278P) and Krt14 (PRB-155P) (both from Covance, Berkeley, CA, USA), Ecad (U3254, Sigma, St Louis, Missouri, USA), Jag1 (sc-8303, H-114, Santa Cruz Biotechnology, Santa Cruz, CA, USA), Krt 5 (905901, Biolegend, San Diego, CA, USA), N1-ICD (#4147, Cell signaling Technology, Boston, MA, USA)

### Quantitative-PCR

3T3/HeLa cells were cultured in Dulbecco's Modified Eagle's Medium supplemented with 10% fetal bovine serum and 1% penicillin/streptomycin (Invitrogen) at 37°C. For the *Six1* knockdown, transient transfection of *Six1* siRNA (s73792, Ambion, Carlsbad, CA) was performed in 3T3 cells. Total RNA from 3T3 cells was extracted using the MiniRNA Plus kit (Qiagen, Hilden, Germany) and converted to cDNA using the High-Capacity cDNA Reverse Transcription Kit (Applied Biosystems, Foster City, CA). Quantitative-PCR was performed using the PCR SYBR green 2X master mix (Invitrogen, Carlsbad, CA) in the StepOne plus Real time PCR instrument [46]. For the Six2 knockdown, transient transfection of *Six2* cDNA (clone TCM1304, Transomic, Huntsville, AL) was performed with Lipofectamine 3000 (cat#L3000015, Invitrogen, Carlsbad, CA) according to the manufacturer's instruction. After 18 hours, cells were transfected with *Six2* siRNA (Silencer Select, s73794, Ambion, Carlsbad, CA) or scrambled siRNA with a final concentration of 20 nM using RNAi Max (cat#13778150, Invitrogen, Carlsbad, CA) according to the manufacturer's instruction. siRNA silencing was conducted a second time after 8 hours. Cells were collected for Quantitative-PCR analysis following the *Six2* cDNA overexpression for 48 hours and the *Six2* siRNA knockdown for 24 hours. The primer sequences used were: *Six1*: 5’- ATGCTGCCGTCGTTTGGTT -3’, 5’-CCTTGAGCACGCTCTCGTT -3’, *Six2*: 5’- CACCTCCACAAGAATGAAAGCG-3’, 5’-CTCCGCCTCGATGTAGTGC -3’, *Gapdh*: 5’-AGGTCGGTGTGAACGGATTTG-3’, 5’-TGTAGACCATGTAGTTGAGGTCA-3’.

### Chromatin immunoprecipitation

P4 old lacrimal glands collected from 40 mouse pups were incubated with 1ml trypsin for 5 minutes and pipetted a few times to dissociate into single cells. 4ml DMEM+10% FBS was added to neutralize the trypsin before addition of 270μl formaldehyde (37%) in 10ml of DMEM containing 10% FBS to fix the cells with shaking for 10 minutes. The cross linking was stopped by addition of DMEM with 10% FBS and 0.125M glycine for 5 minutes. After washed with cold 1xPBS twice (5 minutes each) and centrifuged in 3000 rpm for 3 minutes, the cells were collected and re-suspended in 1ml of ChIP lysis buffer (10mM Tris-Cl, pH8, 85mM KCl, 0.5% NP-40, 5nM EDTA, 0.25% Triton; RIPA- 1% Triton, 150mM NaCl, 0.1% SDS, 0.1% Na-Deoxycholate, 10mM Tris-Cl, pH8, 5mM EDTA) with 1X protease inhibitor and kept in rocker at 4°C for 10 minutes. The cells were spun at 3K rpm, re-suspended with 1ml of RIPA buffer with 1X protease inhibitor before being sonicated with the power 1 second “on”, 2 second “off” for 8 minutes and spun in 15000 rpm for 10 minutes at 4°C. Pre-cleared by incubating with 45 μl agarose beads for 2 hrs at 4°C, the supernatant was spun at 3K rpm and incubated overnight with 1μg of antibody for 1mg of protein at 4°C, followed by 20 μl protein G bead for 2 hrs. The beads were washed with RIPA, Wash buffer A (50mM HEPES, pH7.9, 500mM NaCl, 1mM EDTA, 1% Triton, 0.1% Na-deoxycholate, 0.1% SDS), wash buffer B (20mM Tris-Cl, pH8, 1mM EDTA, 250 mM LiCl, 0.5% NP-40, 0.5% Na-deoxycholate) and TE buffer twice respectively (5 min each wash). After they were spun at 2000 rpm, the collected beads were incubated with 480μl elution buffer (1% SDS, 30mM Tris-Cl (pH8), 15mM EDTA, 200mM NaCl) at 50°C overnight before adding the same volume of phenol:chloroform and centrifuging at 15000 rpm for 5 minutes. The supernatant was mixed with 2X ethanol (100%) at -80°C for 30 minutes, recovered to RT and centrifuged at 15000 rpm at 4°C for 15 minutes. The pellet was washed with 70% ethanol and the air dried DNA was dissolved in 15μl of distilled water for PCR reaction. The mouse monoclonal antibody against Erm was from Proteintech (Catalog number: 66657-1-Ig). The primers used are: CAGCGACTGGAATGAGAACA and GCTGGAACAGGTTGTGTTGA for *Dusp6*, ACTTGGGACTGCCACACTG and AACAACCCCCTCCCTTCTAA for *Col2a1*, TACGATGATGACCGGAAGTG and AGGTTGTTCCAGGTCAGGTG for *Mmp2*, AGTCCCGCTTGATACCTTGA and GTGGCTTTCTCGCTGTCTTT for *Ext1*.

### Lacrimal gland organ culture

The lacrimal glands from E16.5–17.5 embryos were harvested and gently transferred onto filter paper (0.45 um) in 35 mm low bottom dishes (ibidi, Martinsried, Germany) in medium (DMEM, 5%FBS, 400ng/ml Fgf10, 250ng/ml Heparin, 1X ITS, P/S) containing either scrambled, Cy3-labeled negative control (AM4621, Invitrogen, Carlsbad, CA), *Six1* (s73792) or *Six2* (s73794) siRNA. Lipoefectamine-siRNA complexes were prepared in Optimem medium as per the manufacturer’s instructions. To test the genetic redundancy, 45nM of scrambled siRNA, 15nM Six1 + 30nM scrambled siRNA, 30nM Six2 +15nM scrambled siRNA and 15nM Six1 + 30nM Six2 siRNA were used. 10μl of matrigel and medium in a 1:1 ratio was added on top of each gland. The glands were cultured for 24–48 hrs at 37°C.

### Laser capture microdissection, RNA sequencing and Bioinformatics analysis

Laser capture microdissection and RNA sequencing were performed as previously described [29]. The RNAseq data is available at the GEO repository under accession number GSE114509. Unsupervised clustering analysis was performed in MATLAB using the Clustergram function. We determined interquartile ranges of the gene expression levels in all samples and the top 200 genes were plotted. GSEA was performed using MATLAB implementation of the same method as described [20]. KEGG pathway enrichment analysis and functional annotation was performed in DAVID. For the functional annotation of downregulated genes, a list of 476 genes was used for the analysis based on cutoff points for the normal expression levels (> 50 units), Log2 (fold change) (<-1) and p-values (<0.05). Volcano plots representing Log2(p-value) vs Log2(fold change) were plotted in MATLAB. -Log2(p-value) > 50 were set to 50 in order to avoid the scaling issues in the plot.

ChIP-seq analysis was performed using MACS [47]. SRA files of ETV1 (ER81), ETV4 (PEA3) and ETV5 (ERM) ChIP-seq data were retrieved from the GEO database [21, 22]. SRA files were converted to a Fastq format using sratoolkit, followed by mapping of the sequence reads on the genome (hg18) to generate a SAM file. Peak calling was done using MACS (using default parameters). The mapped ChIP-seq file was visualized on the human reference genome assembly (hg18) using the UCSC genome browser. The Erm binding sites in the mouse genome were scanned using MATCH algorithm based on TRANSFAC database.

## Supporting information

S1 Fig Chromatin immunoprecipitation confirmed Erm target genes in the lacrimal gland cells. (**A**) Locations of Erm binding sites (blue bars) identified by the TRANSFAC database in *Ext1*, *Dusp6*, *Col2a1* and *Mmp3* mouse genes overlaid with the conservation plot of placental mammal genomes. (**B**) Chromatin immunoprecipitation using anti-Erm antibody showed selective enrichment of *Ext1*, *Dusp6*, *Col2a1* and *Mmp3* sequences compared to IgG antibody control.
(TIF)

S2 Fig Normal lacrimal gland development in the *Six2*^*-/-*^ mutant. (**A-B**) Hematoxylin and Eosin (H&E) and Pax6/Ecad staining showed comparable lacrimal gland budding between the E15.5 control and *Six2*^*-/-*^ mutant embryos (dotted lines).
(TIF)

S3 Fig *Lfng* knockout resulted in up regulation of Notch signaling and lacrimal gland hypoplasia. (**A-D**) Notch-ICD was elevated in the tip of the *Lfng* mutant lacrimal gland bud at E14.5. C and D are the enlarged images of the areas marked in A and B. Arrows point to the tips of the lacrimal gland buds. (**E-F**) At P10, the lacrimal gland in the *Lfng* mutant (F) was reduced in size compared to the wild type control (E).
(TIF)
