# Supplementary figures and images for: FGF-induced Pea3 transcription factors program the genetic landscape for cell fate determination
Source: PLoS Genet. 2018 Sep 6;14(9):e1007660. doi: 10.1371/journal.pgen.1007660 (PMC6143274; doi:10.1371/journal.pgen.1007660)

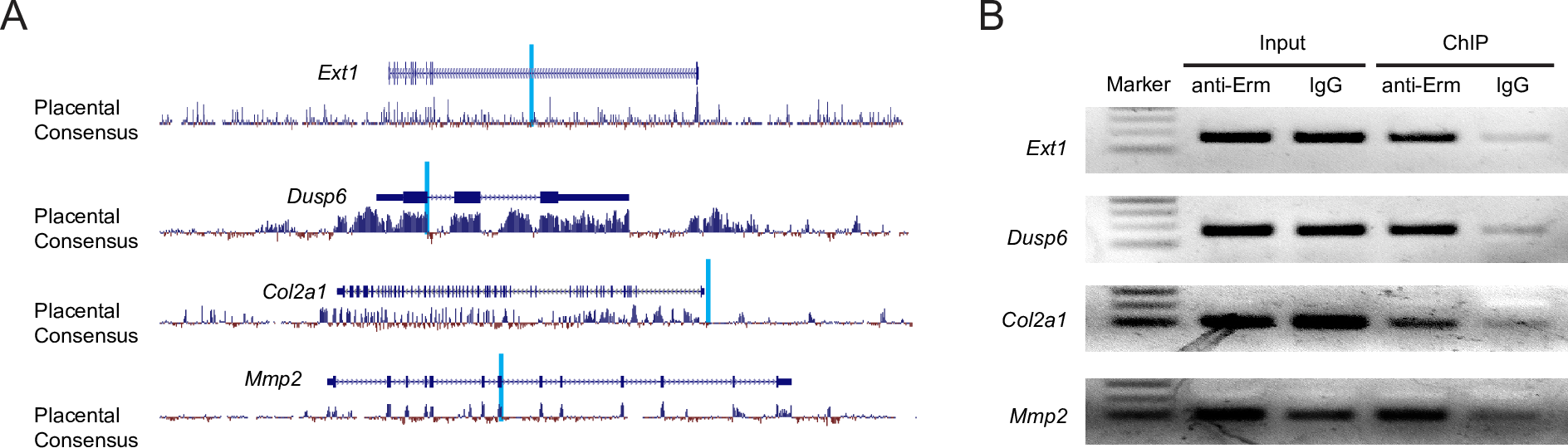

Supplement: S1 Fig — (A) Locations of Erm binding sites (blue bars) identified by the TRANSFAC database in Ext1, Dusp6, Col2a1 and Mmp3 mouse genes overlaid with the conservation plot of placental mammal genomes. (B) Chromatin immunoprecipitation using anti-Erm antibody showed selective enrichment of Ext1, Dusp6, Col2a1 and Mmp3 sequences compared to IgG antibody control. (TIF) [file pgen.1007660.s001.tif]

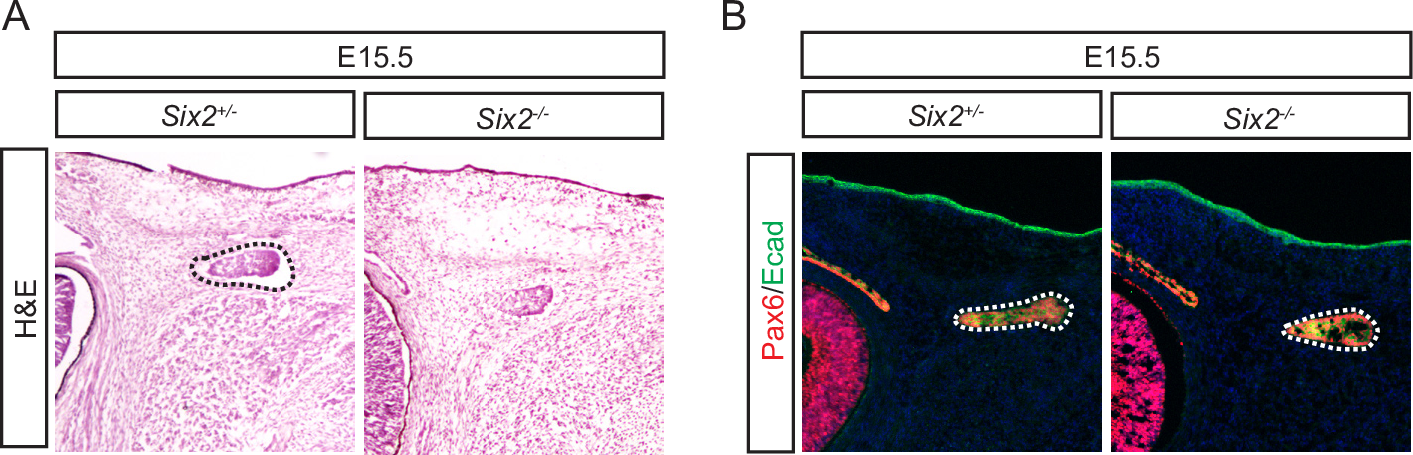

Supplement: S2 Fig — (A-B) Hematoxylin and Eosin (H&E) and Pax6/Ecad staining showed comparable lacrimal gland budding between the E15.5 control and Six2-/- mutant embryos (dotted lines). (TIF) [file pgen.1007660.s002.tif]

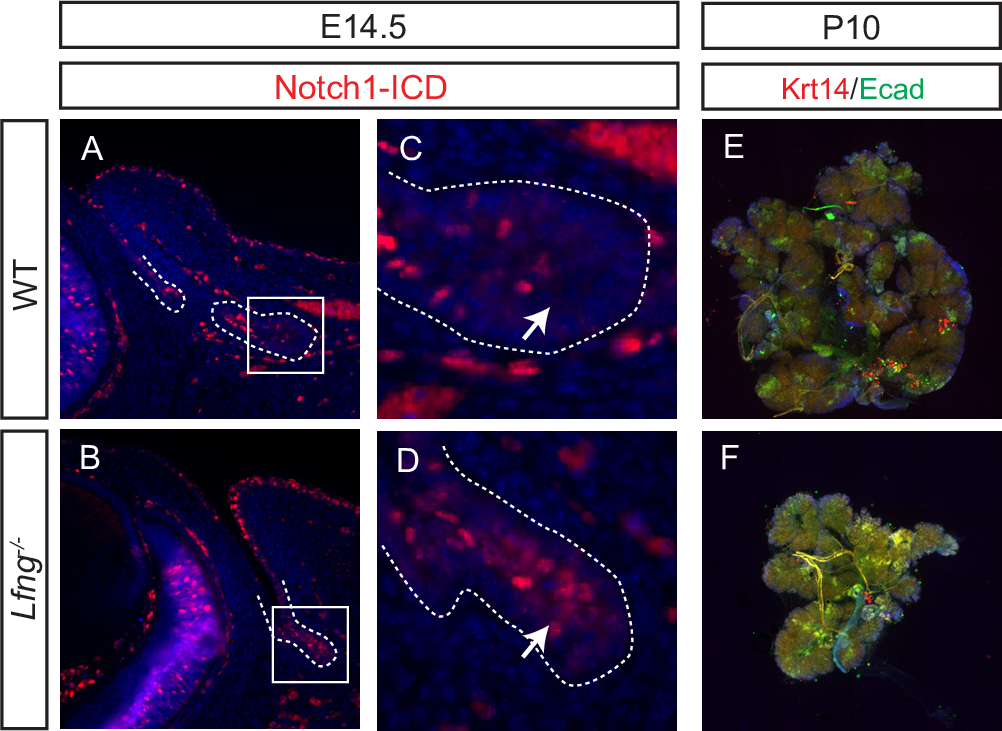

Supplement: S3 Fig — (A-D) Notch-ICD was elevated in the tip of the Lfng mutant lacrimal gland bud at E14.5. C and D are the enlarged images of the areas marked in A and B. Arrows point to the tips of the lacrimal gland buds. (E-F) At P10, the lacrimal gland in the Lfng mutant (F) was reduced in size compared to the wild type control (E). (TIF) [file pgen.1007660.s003.tif]
